# Microbiological Profile of Periprosthetic Infections Following Femoral Fracture: A Retrospective Analysis

**DOI:** 10.3390/jcm15103744

**Published:** 2026-05-13

**Authors:** Luca Bianco Prevot, Edoardo Verme, Livio Pietro Tronconi, Francesco Busardò, Giuseppe Basile

**Affiliations:** 1 Department of Biomedical Sciences and Public Health, University “Politecnica Delle Marche” of Ancona, 60124 Ancona, Italy; l.biancoprevot@pm.univpm.it (L.B.P.); f.busardo@univpm.it (F.B.); g.basile@univpm.it (G.B.); 2 Residency Program in Orthopedics and Traumatology, University of Milan, Via Festa del Perdono 7, 20122 Milan, Italy; 3 Department of Health and Life Sciences, European University of Rome, 00163 Rome, Italy; liviopietro.tronconi@unier.it; 4 Research Center of Legal Medicine and Risk Management, Cardiovascular Department, Maria Cecilia Hospital, GVM Care & Research, 48033 Cotignola, Italy

**Keywords:** femoral fracture, periprosthetic joint infection, implant-related infection, geriatric trauma, microbiology

## Abstract

**Background**: Implant-related infection following femoral fracture surgery is a severe complication in elderly patients and is associated with high morbidity and mortality. Most available evidence on periprosthetic joint infection (PJI) derives from elective arthroplasty populations, which differ substantially from patients undergoing surgery for femoral fractures. This study aimed to investigate the microbiological profile and clinical characteristics of implant-related infections after proximal femoral fracture surgery. **Materials and Methods**: A retrospective observational study was conducted on 20 patients aged ≥70 years who developed implant-related infection after surgical treatment of proximal femoral fractures between 2020 and 2025 at a referral trauma center. Surgical procedures included intramedullary nailing, hemiarthroplasty, and total hip arthroplasty. Only patients with Charlson Comorbidity Index ≥ 4 and infection occurring within one year of the index surgery were included. Clinical, surgical, microbiological, and antibiotic therapy data were retrospectively reviewed. **Results**: The cohort had a mean age of 82.4 years and a high comorbidity burden (mean Charlson index 4.8). The most frequently isolated pathogen was *Staphylococcus aureus* (25.9%), with 85% methicillin-resistant strains. Other pathogens included *Enterococcus faecalis*, *Klebsiella pneumoniae*, and *Escherichia coli*. Polymicrobial infections were observed in 25% of patients. One-year mortality was 25%. **Conclusions**: Implant-related infections after femoral fracture surgery represent a distinct clinical entity compared with elective PJI, characterized by frail patients and a higher prevalence of multidrug-resistant organisms. These findings highlight the need for tailored preventive and therapeutic strategies in this high-risk population.

## 1. Introduction

Femoral fractures are among the most common injuries in geriatric trauma and carry a high burden in terms of mortality, functional decline, and healthcare utilization [1]. Surgical treatment, whether total hip arthroplasty, hemiarthroplasty, or intramedullary nailing is almost always required and is performed in patients who are typically elderly, frail, multimorbid, and exposed to prolonged hospitalization [2]. In this vulnerable population, implant-related infection represents one of the most serious complications.

Among these complications, the most feared is periprosthetic joint infection (PJI), a challenging condition to manage and one that carries a poor prognosis in terms of both functional recovery and overall survival often unavoidable despite the preventive measures currently in place [3,4,5]. Most of the existing knowledge on PJI, particularly regarding microbiology, antibiotic prophylaxis, and therapeutic strategies, derives from studies on elective arthroplasty, which typically involve younger and healthier patients whose characteristics differ substantially from those treated for femoral fractures [6,7]. Such differences in patient profile, urgency of surgery, and perioperative circumstances may significantly influence pathogen distribution, antimicrobial resistance patterns, and clinical outcomes [8]. Indeed, microbiological studies have shown that infections in frail or hip-fracture patients often involve more aggressive or resistant organisms, with a higher prevalence of Gram-negative bacteria and polymicrobial infections compared with elective PJI [9]. Despite this, few studies provide a comprehensive assessment of implant-related infections across the different surgical approaches used to treat femoral fractures. Evidence remains fragmented and often limited to a single technique, without evaluating whether current antibiotic prophylaxis protocols derived largely from elective arthroplasty are appropriate for this high-risk population.

To address these gaps, the present study investigates implant-related infections in patients treated with arthroplasty, endoprosthesis, or intramedullary nailing for femoral fractures. The aim is to characterize microbial epidemiology, antibiotic susceptibility patterns, and the alignment between administered prophylaxis and isolated pathogens, thereby informing more appropriate preventive and therapeutic strategies tailored to geriatric trauma.

## 2. Materials and Methods

The present study focuses on 20 patients affected by periprosthetic joint infection occurring after proximal femoral fracture, treated at the Traumatology Division of IRCCS Galeazzi–S. Ambrogio, Milan, Italy, between 2020 and 2025. Clinical records, operative notes, and laboratory data were retrospectively reviewed. This observational retrospective study was conducted on a cohort of patients who sustained a femoral fracture complicated by PJI. Data were obtained from medical charts, surgical and microbiological documentation, and available follow-up evaluations.

Only patients aged 70 years or older were included, provided that they had undergone surgical treatment of a femoral fracture with intramedullary nailing, hemiarthroplasty, or total hip arthroplasty, and had subsequently developed an implant-related infection within one year of the index surgery. Inclusion also required a substantial comorbidity burden, documented by a Charlson Comorbidity Index score of 4 or higher. Patients were excluded if they were younger than 70 years, had no significant comorbidities, or had undergone revision procedures unrelated to infection before the infectious episode. Additional exclusions involved cases with incomplete documentation or those in which the interval between the primary procedure and the infection-related surgery could not be reliably determined.

PJI was defined according to the criteria described by Parvizi et al., requiring at least one of the following: the presence of a sinus tract communicating with the prosthesis; isolation of the same microorganism from two or more cultures or tissue biopsies obtained from the affected joint; or isolation of a single microorganism from intraoperative cultures associated with additional evidence of implant infection, such as positive histology, purulence, or elevated inflammatory markers including erythrocyte sedimentation rate, C-reactive protein, and synovial white blood cell count.

Infections were classified based on the time elapsed between surgery and the onset of symptoms, following the modified criteria proposed by Tsukayama et al. Early postoperative infections occur within the first four weeks after surgery and are typically caused by intraoperative contamination, presenting acute symptoms such as pain, redness, and wound drainage. Late postoperative infections develop more than four weeks after implantation and are often due to low-virulence microorganisms, resulting in a more subtle but persistent clinical picture, frequently characterised by chronic pain and implant loosening. A third category includes hematogenous infections, which arise when bacteria spread to the prosthesis through the bloodstream from a distant infectious focus. These infections may appear suddenly, even years after surgery, in a previously well-functioning joint.

For each patient, all relevant clinical, demographic, surgical, and microbiological data were systematically collected to define the individual risk profile and the clinical course of the infection. Age, sex, medical history, and comorbidities were recorded, and the Charlson Comorbidity Index was calculated as an essential inclusion parameter. The date and type of primary surgery for the femoral fracture were documented, distinguishing among intramedullary nailing, hemiarthroplasty, and total hip arthroplasty, along with the date of the first procedure performed in relation to the onset of infection. Information regarding preoperative antibiotic prophylaxis administered at the time of the index surgery was obtained, together with details of the antimicrobial therapy prescribed after the diagnosis of infection. All cultured microorganisms were identified, with specific attention to antimicrobial susceptibility and resistance patterns, including the presence of multiple isolates or polymicrobial infections. Additionally, all available data concerning the clinical course, perioperative and postoperative complications, need for further revision procedures, follow-up outcomes, and mortality within one year of surgery were reviewed. Informed consent was obtained from all patients.

## 3. Results

The study population included 20 patients, with an equal sex distribution of 50% female and 50% male. The median age was 82.4 years (SD 6.8). Patients admitted from rehabilitation facilities, nursing homes, hospitals, or long-term care institutions were 60%. Comorbidity burden was substantial, with a mean Charlson Comorbidity Index of 4.8 (SD 1.2). All patients presented multiple comorbidities, the most frequent being arterial hypertension (22.4%), type 2 diabetes (13%), previous myocardial infarction, and dementia. Twenty-five percent of the patients died within one year of follow-up. General patient characteristics are summarized in Table 1.

Half of the infections occurred in patients who had undergone hemiarthroplasty implantation (10 patients), 30% in those with total joint arthroplasty (6 patients), and 20% in patients treated with intramedullary nailing for fracture fixation (4 patients). All prosthetic implants were non-cemented. The median time between the initial fracture treatment and revision surgery was 89.3 days (SD 69.18). The main surgical procedure for PJI management was debridement, antibiotics, and implant retention (DAIR), performed in 35% of cases, followed by implant or hardware removal with placement of an antibiotic-loaded cement spacer in 60% of cases, and by Girdlestone resection arthroplasty in the remaining 5%.

About ASA classification, one patient (5.9%) was classified as ASA I, seven (41.2%) as ASA II, and nine (52.9%) as ASA III. The mean surgical length of the index procedure was 61.3 min (SD 22.8).

The most frequently isolated pathogen was Staphylococcus aureus, identified in 25.9% of patients, 85% of which were methicillin-resistant strains (MRSA). Other pathogens included Enterococcus faecalis (14.8%), Klebsiella pneumoniae (11.1%, all ESBL-producing), Escherichia coli (11.1%), Staphylococcus epidermidis (7.4%), Staphylococcus capitis (7.4%), Candida albicans (3.7%), Proteus mirabilis (3.7%), Pseudomonas aeruginosa (3.7%), and Streptococcus pyogenes (3.7%). Detailed resistance profiles of the isolated microorganisms are presented in Table 2.

Polymicrobial infection was observed in 25% of patients. The mean duration of antibiotic therapy was 4.5 weeks (SD1.8). Details regarding the antibiotic regimens used are reported in Table 3.

## 4. Discussion

Our findings indicate that infections occurring after proximal femoral fracture repair present a microbiological profile that differs substantially from conventional elective PJI, with a higher prevalence of Gram-negative and multidrug-resistant organisms. Many of the pathogens isolated were resistant to cefazolin, which remains the standard prophylactic agent in hip-fracture surgery, highlighting a clear mismatch between current preventive protocols and the actual microbial landscape in this high-risk population. These observations suggest that perioperative prophylaxis may need to be reconsidered, potentially incorporating alternative agents capable of providing broader and more appropriate coverage. Given the increasing circulation of resistant organisms and the considerable morbidity, mortality, and healthcare burden associated with these infections, dedicated strategies for prevention and management should be further investigated and refined.

This study cohort was characterized by advanced age and a high comorbidity burden, as indicated by a mean Charlson Comorbidity Index of 4.8. This aligns with the literature, which consistently reports that hip-fracture patients frequently present multimorbidity, impaired immune function, and reduced physiological reserve factors that increase susceptibility to infection and significantly worsen clinical outcomes [10,11]. The one-year mortality rate of 25% observed in our series further underscores the vulnerability of this population and mirrors previously reported mortality rates following periprosthetic hip infection in geriatric trauma patients [12,13]. Infectious complications occurred across all fracture-treatment strategies, including hemiarthroplasty, total hip arthroplasty, and intramedullary nailing. This distribution highlights that no implant type is exempt from severe infectious complications when used in frail, elderly, high-risk patients [14]. The median interval of 89 days between the index procedure and revision surgery reflects the heterogeneity of clinical presentation, encompassing both early and late infections, in line with the Tsukayama classification. The microbiological profile observed in this study shows substantial differences from that expected in elective PJI. Staphylococcus aureus was the most frequently isolated pathogen in our cohort, and notably, 85% of isolates were methicillin-resistant (MRSA). This proportion is substantially higher than that reported in the current literature, where MRSA typically accounts for approximately 20% of *S. aureus*–associated PJI [15], and likely reflects the clinical complexity of frail elderly patients, characterized by repeated hospitalizations, cumulative antibiotic exposure, and frequent transitions between acute-care and long-term-care facilities, in contrast to elective arthroplasty populations. A considerable proportion of infections was caused by Gram-negative bacteria, including extended-spectrum β-lactamase–producing *Klebsiella pneumoniae*, *Escherichia coli*, and *Pseudomonas aeruginosa*. In addition, *Enterococcus faecalis* accounted for 14% of infections in our cohort, a prevalence markedly higher than the approximately 5% generally reported in the literature for periprosthetic joint infections [7]. As an opportunistic pathogen, *Enterococcus faecalis* is commonly associated with frail patients affected by multiple comorbidities, prolonged hospitalization, and prior antibiotic exposure. The presence of *Candida albicans* and a 25% rate of polymicrobial infections further underscore the complexity and severity of infection patterns observed in geriatric trauma, reflecting a microbial landscape that differs substantially from that typically described in elective arthroplasty. These findings raise important considerations regarding the adequacy of current perioperative antibiotic prophylaxis. Protocols used for femoral-fracture surgery generally mirror those used in elective arthroplasty and rely primarily on cefazolin [16,17,18]. However, many of the microorganisms isolated in our cohort demonstrated resistance to first-line cephalosporins, and a substantial proportion consisted of MRSA and multidrug-resistant Gram-negative organisms. This discrepancy suggests that standard prophylaxis may be insufficient to prevent infection in elderly, frail, multimorbid patients. In the current literature, only a limited number of studies have explored alternative prophylactic approaches in this setting, such as the use of intra-articular vancomycin administration in patients undergoing hemiarthroplasty for femoral fracture [19] or comparisons between perioperative prophylaxis with cefazolin and vancomycin [20]. These studies are generally characterized by small sample sizes and limited statistical power, resulting in inconclusive findings; nevertheless, their results support the need to generate new evidence aimed at optimizing infection-prevention strategies in high-risk populations. Future research should therefore focus on well-designed studies to better define the role of alternative prophylactic regimens while adhering to antimicrobial stewardship principles. From a therapeutic standpoint, the management of PJI in this population is particularly challenging. Although DAIR procedures accounted for 35% of cases, implant removal with placement of an antibiotic-loaded cement spacer was required in 60%, underscoring the severity of these infections and the frequent need for more aggressive surgical interventions. Multimorbidity and limited surgical tolerance further complicate decision-making and often constrain the therapeutic options available. The ASA score distribution of our cohort, with more than half of patients classified as ASA III, is consistent with the existing literature identifying elevated ASA classification as an independent predictor of PJI, reflecting the compounded effect of systemic comorbidities on immune response and resistance to microbial colonization [21]. Operative time, with a mean of 61.3 min in our series, represents an additional recognized risk factor: prolonged surgical exposure is associated with increased bacterial contamination and reduced efficacy of antibiotic prophylaxis [22]. Although this duration does not exceed the threshold requiring prophylaxis redosing, it must be interpreted in the context of a population already burdened by high comorbidity, where even standard operative times may carry a disproportionate infectious risk.

### Medical Legal Considerations

In the surgical treatment of proximal femoral fractures in elderly patients, periprosthetic joint infection should not be interpreted from a medico-legal perspective as an exceptional or anomalous event, but rather as an adverse outcome with a high conditional probability, structurally linked to the unavoidable need for urgent surgical treatment, the biological vulnerability of the host, and the intrinsic, scientifically documented limits of modern medicine in achieving complete control of infection risk associated with the implantation of foreign materials [23]. In this context, the surgical act is characterized by a clinically determined decisional constraint, in which the surgeon’s discretionary margin is physiologically compressed by therapeutic urgency, the limited practicability of effective alternatives, and the primary need to preserve patient survival, rendering the automatic application of evaluative paradigms derived from elective surgery methodologically inadequate [24]. The predictability of infection therefore assumes a predominantly statistical-epidemiological connotation, based on evidence demonstrating that advanced age, multimorbidity, institutionalization, and emergency surgery represent independent determinants of a significantly increased infectious risk, configuring a known, measurable, and not fully eliminable risk [25]. Consequently, the assessment of healthcare liability cannot be grounded on an outcome-based criterion, but must instead be anchored to the rationality of the ex ante decision-making process, to the appropriateness and proportionality of preventive measures adopted in relation to the patient’s real risk profile, and to the adequacy of organizational and managerial choices, thereby shifting the axis of attribution from individual technical fault toward a predominantly systemic responsibility focused on the healthcare system’s capacity to recognize, integrate, and govern a structurally elevated risk [26,27]. In this perspective, host-related biological factors, particularly immunosenescence, multimorbidity, and malnutrition, assume decisive relevance as autonomous causal contributors in the determinism of infectious events, requiring probabilistic and multifactorial causal models and a conception of preventability in terms of reasonable risk reduction rather than absolute elimination [28,29]. This framework also entails a renewed centrality of personalized informed consent, which must concretely and specifically represent the real infectious risk in fragile patients, under penalty of autonomous liability for violation of the right to self-determination [30], as well as a dynamic and probabilistic evaluation of damage according to the loss-of-chance paradigm [31], delineating an advanced medico-legal model in which periprosthetic joint infection after hip fracture represents a paradigmatic expression of complex and systemic clinical risk rather than individual technical error.

## 5. Conclusions

Overall, our findings demonstrate that implant-related infections after proximal femoral fracture constitute a distinct clinical entity compared with elective PJI. The combination of patient frailty, highly resistant pathogens, and complex surgical management underscores the need for dedicated preventive and therapeutic protocols. Larger prospective studies are required to better define prophylactic strategies and to optimize diagnostic and therapeutic algorithms for this extremely high-risk population.

## Figures and Tables

**Table 1:** Characteristics of the study population.

N°	AGE	Sex	CCH	Comorbidity	1° Surgery	ASA	Surgical Length	1° LOS	1° Surgery Complications	Time Between 1° and 2° Surgery	2° Surgery	2° Surgery Complications	2° Surgery LOS		1 Year Death
1	77	M	5	HTN, prostate cancer, anal stenosis	arthroplasty	2	73	4	no	236	cement spacer implantation		no	17	no
2	80	F	6	HTN, type 2 diabetes, COPD, atrial fibrillation	arthroplasty	3	110	30	UTI	93	cement spacer implantation		no	25	no
3	93	F	6	HTN, type 2 diabetes, lower-limb thrombophlebitis	intramedullary nail	3	35	8	postoperative anemia	61	cement spacer implantation		no	23	yes
4	79	F	4	HTN, myocardial Infarction	hemiarthroplasty	2	90	6	no	66	cement spacer implantation		spacer dislocation	34	no
5	82	M	6	COPD, previous TIA, hypertensive heart disease, anemia, HTN	hemiarthroplasty	3	50	12	postoperative anemia	39	DAIR		pneumonia	30	no
6	92	M	7	Multiple strokes, right-leg amputation, dementia, HTN	hemiarthroplasty	/	/	/	no	140	DAIR		no	23	yes
7	86	M	4	HTN, obesity, osteoporosis, hypothyroidism	hemiarthroplasty	3	60	21	no	44	cement spacer implantation		postoperative anemia	60	no
8	70	M	4	HTN, obesity, hypothyroidism	arthroplasty	2	60	6	no	59	cement spacer implantation		spacer dislocation	44	no
9	81	M	5	Hyperthyroidism, chronic liver disease, acute pancreatitis, dementia	arthroplasty	/	/	/	no	183	cement spacer implantation		spacer dislocation + postoperative anemia	50	no
10	73	F	4	type 2 diabetes	intramedullary nail	3	24	5	postoperative anemia	23	DAIR		no	20	no
11	87	M	5	Hypertension, glaucoma, Parkinson’s disease, depression, vascular encephalopathy	hemiarthroplasty	/	/	/	no	38	cement spacer implantation		postoperative anemia		no
12	80	F	4	HTN, myocardial Infarction	hemiarthroplasty	3	60	10	hyponatremia, postoperative anemia	262	cement spacer implantation		Spacer dislocation	30	no
13	88	F	7	Chronic venous insufficiency, HTN, diabetes, Alzheimer’s disease	hemiarthroplasty	3	90	7	postoperative anemia, pressure sores	12	DAIR		pneumonia	33	no
14	86	F	5	TIA, HTN, GERD	hemiarthroplasty	3	40	5	no	4	cement spacer implantation		no	7	yes
15	91	M	5	Cognitive impairment, dermatitis, HTN, ocular herpes	hemiarthroplasty	3	60	7	postoperative anemia	45	cement spacer implantation		no	29	yes
16	87	F	6	Single kidney, lung cancer	intramedullary nail	2	35	5	postoperative anemia	94	DAIR		no	15	no
17	88	F	4	Single kidney, lung cancer	intramedullary nail	2	45	7	postoperative anemia	51	DAIR		pneumonia + pleural effusion	23	yes
18	76	M	4	Stroke	hemiarthroplasty	2	43	5	postoperative anemia	34	DAIR		no	19	no
19	82	F	4	HTN, anxiety–depressive syndrome	arthroplasty	2	90	10	no	25	Gilderstone		postoperative anemia	18	no
20	70	M	3	HTN, hepatitis	arthroplasty	1	75	3	no	278	cement spacer implantation		no	10	no

hypertension (HTN), transient ischemic attack (TIA), gastroesophageal reflux disease (GERD), chronic obstructive pulmonary disease (COPD), urinary tract infection (UTI), debridement antibiotic and implant retention (DAIR).

**Table 2:** Characteristics of perioperative and postoperative antibiotic prophylaxis following fracture treatment, and antibiotic therapy administered after revision surgery for infection and after culture results.

N°	Perioperative Antibiotic Prophylaxis	Post-Operative Antibiotic Prophylaxis	Pre-Culture Antibiotic Therapy	Post-Culture Antibiotic Therapy (Inpatient)	Antibiotic Therapy After Post-Culture (Home Treatment)
1	ceftriaxone 2 g	cefazolin 2 g daily × 4 days	vancomycin 2 g + piperacillin/tazobactam 13.5 g daily	ampicillin 4 g daily	amoxicillin/clavulanate 3 g daily (3 weeks)
2	cefazolin 2 g	cefazolin 2 g daily × 4 days	meropenem 3 g daily	meropenem 3 g + daptomycin 700 mg + caspofungin 50 mg daily	meropenem 3 g + daptomycin 700 mg + caspofungin 50 mg daily/4 weeks)
3	cefazolin 2 g	cefazolin 2 g daily × 5 days	vancomycin 2 g + piperacillin/tazobactam 13.5 g daily	vancomycin 2 g + piperacillin/tazobactam 13.5 g daily	†
4	cefazolin 2 g	cefazolin 2 g daily × 5 days	vancomycin 2 g + piperacillin/tazobactam 13.5 g daily	cephalexin 6 g daily	cefalexin 6 g daily (4 weeks)
5	cefazolin 2 g	cefazolin 2 g daily × 5 days	vancomycin 2 g + piperacillin/tazobactam 13.5 g daily	vancomycin 2 g + rifampicin 600 mg	vancomycin 2 g + rifampicin 600 mg daily (4 weeks)
6	/	/	vancomycin 2 g + meropenem 3 g daily	vancomycin 2 g + meropenem 3 g daily	meropenem 3 g daily (4 weeks)
7	levofloxacin 500 mg + vancomycin 500 mg	levofloxacin 500 mg + vancomycin 500 mg × 3 days	ciprofloxacin 800 mg die + meropenem 3 g daily	vancomycin 2 g + meropenem 3 g daily	vancomycin 3 g daily (4 weeks)
8	cefazolin 2 g	cefazolin 3 g daily × 5 days	levofloxacin 500 mg + vancomycin 3 g daily	levofloxacin 750 mg + vancomycin 3 g daily	amoxicillin/clavulanate 3 g daily (4 weeks)
9	/	/	vancomycin 1 g + meropenem 1 g daily	amikacin 500 mg + ciprofloxacin 500 mg daily	ciprofloxacin 500 mg daily (4 weeks)
10	cefazolin 2 g	cefazolin 3 g daily × 5 days	vancomycin 2 g + meropenem 3 g daily	vancomycin 2 g + meropenem 3 g daily	trimethoprim/sulfamethoxazole 1.9 g daily (4 weeks)
11	/	/	vancomycin 2 g daily	meropenem 3 g + daptomycin 500 mg daily	meropenem 3 g + doxycycline 200 mg daily (8 weeks)
12	cefazolin 2 g	cefazolin 3 g daily × 5 days	vancomycin 3 g + levofloxacin 500 mg daily	ampicillin/sulbactam 3 g daily	†
13	cefazolin 2 g	cefazolin 3 g daily × 5 days	levofloxacin 1 g daily	daptomycin 700 mg + doxycycline 200 mg daily	doxycycline 200 mg daily (4 weeks)
14	cefazolin 2 g	cefazolin 2 g daily × 5 days	piperacillin/tazobactam 13.5 g daily	teicoplanin 600 mg + piperacillin/tazobactam 13.5 g daily	teicoplanin 600 mg + piperacillin/tazobactam 13.5 g daily (4 weeks)
15	cefazolin 2 g	cefazolin 2 g daily × 5 days	piperacillin/tazobactam 13.5 g daily	†	†
16	cefazolin 2 g	cefazolin 2 g daily × 5 days	daptomycin 350 mg + piperacillin/tazobactam 13.5 g daily	daptomycin 350 mg + piperacillin/tazobactam 13.5 g daily	trimethoprim/sulfamethoxazole 1.9 g + rifampicin 600 mg daily (10 weeks)
17	cefazolin 2 g	cefazolin 2 g daily × 5 days	linezolid 1.2 g daily	linezolid 1.2 g daily	daptomycin 500 mg daily (4 weeks)
18	cefazolin 2 g	cefazolin 3 g daily × 5 days	ceftriaxone 2 g + clindamycin 200 mg daily	meropenem 3 g + Fosfomycin 16 g daily	meropenem 3 g + Fosfomycin 16 daily (6 weeks)
19	cefazolin 2 g	cefazolin 1 g daily × 5 days	/	/	trimethoprim/sulfamethoxazole 1.9 g daily (4 weeks)
20	cefazolin 2 g	cefazolin 3 g daily × 5 days	levofloxacin 500 mg + vancomycin 1 g	levofloxacin 750 mg + rifampicin 600 mg + clindamycin 1.8 g daily	cefazolin 6 g daily (2 weeks)

/ (no therapy adopted), † (The patient has passed away).

**Table 3:** Types of pathogens and their antibiotic susceptibility profiles (green: Susceptible (S), yellow: Intermediate (I), red: Resistant (R)).

N°	Pathogen I	Pathogen II	Beta-Lactam														Macrolide				Aminoglycoside						Carbapenems				Quinolones				Glycopeptide							
			Ampicillin + Sulbactam		Oxacillin		Piperacillin + Tazobactam		Cefotaxime		Ceftazidime		Cefepime		Clindamycin		Erythromycin		Gentamycin		Amikacin		Ertapenem		Meropenem		Levofloxacin		Ciprofloxacin		Teicoplanin		Vancomycin		Linezolid		Trimethoprim + Sulfamethoxazole		Fosfomycin		Daptomycin	
1	*E. faecalis*	/	S		/		/		/		/		/		R		R		R		/		/		/		R		/		S		S		S		S		/		/	
2	*K. pneumonie*	*C. albicans*	/		/		R		R		R		R		/		/		R		S		S		S		/		R		/		/		/		S		R		/	
3	*K pneumonie*	/	/		/		R		R		R		R		/		/		S		S		S		S		/		R		/		/		/		S		S		/	
4	*S.aureus*	/	/		R		/		/		/		/		S		S		S		/		/		/		I		/		S		S		S		S		/		/	
5	*S.aureus*	/	/		R		/		/		/		/		R		R		S		/		/		/		R		/		S		S		S		S		/		/	
6	*P. mirabilis*	/	/		/		S		R		R		S		/		/		S		S		S		/		/		R		/		/		/		R		R		/	
7	*E. coli*	/	/		/		R		/		/		R		/		/		R		I		/		S		/		/		/		S		/		R		S		/	
8	*S. epidermidis*	/	/		S		/		/		/		/		S		S		S		/		/		/		S		/		S		S		S		S		/		/	
9	*E. coli*	/	/		/		S		S		S		S		/		/		S		S		S		/		/		S		/		/		/		S		S		/	
10	*S. aureus*	/	/		R		/		/		S		/		R		R		S		/		/		/		R		/		S		S		S		S		/		S	
11	*P. auruginosa*	*S. capitis*	/	/	/	R	I	R	/	/	I	/	I	S	S	/	R	R	R	R	S	/	/	/	S	/	R	R	I	/	S	S	S	S	S	S	I	/	/	/	/	/
12	*E. faecalis*	/	S		/		/		/		/		/		R		R		S		/		/		/		S		/		S		S		S		/		/		/	
13	*S. capitis*	/	/		R		/		/		/		/		R		R		R		/		/		/		R		/		S		S		/		S		/		S	
14	*S. epidermidis*	/	/		R		/		/		/		/		S		S		R		/		/		/		R		/		S		S		S		S		/		S	
15	*S.aureus*	*E. faecalis*	/	R	R	/	/	/	/	R	/	/	/	/	R	R	R	R	S	S	/	/	/	/	/	/	R	R	/	/	/	S	/	S	S	S	S	S	/	/	/	S
16	*S.aureus*	/	/		R		/		/		/		/		R		R		S		/		/		/		R		/		S		S		S		S		/		S	
17	*S. aureus*	*S. pyogenes*	/	/	R	/	/		/		/		/	/	R	/	R	/	S	/	/	/	/	/	/	/	R	/	/		/	/	/	/	S	/	S	/	/	/	/	/
18	*K. pneumonie*	/	/		/		R		R		/		I		/		/		S		S		S		S		/		R		/		/		/		S		/		/	
19	*E. coli*	*E. faecalis*	S	R	/	R	/	/	R	R	S	/	S	/	/	R	/	R	S	S	S	/	S	/	/	/	/	R	I	I	S	S	S	S	S	S	S	/	/	/	/	/
20	*S. aureus*	/	/		S		S		/		/		S		S		/		/		/		/		S		/		S		S		/		/		S		/		/	

## Data Availability

The data supporting the findings of this study are available from the corresponding author upon reasonable request.
